# Optical tweezing and binding at high irradiation powers on black-Si

**DOI:** 10.1038/s41598-017-12470-9

**Published:** 2017-09-26

**Authors:** Tatsuya Shoji, Ayaka Mototsuji, Armandas Balčytis, Denver Linklater, Saulius Juodkazis, Yasuyuki Tsuboi

**Affiliations:** 1 Division of Molecular Materials Science, Graduate School of Science, Osaka City University, 3-3-138 Sugimoto, Sumiyoshi, Osaka, 5558-8585 Japan; 2 Centre for Micro-Photonics, Swinburne University of Technology, John Street, Hawthorn, 3122 Vic Australia; 3 Center for Physical Sciences and Technology, A. Goštauto 9, LT-01108 Vilnius Lithuania; 4 Melbourne Centre for Nanofabrication, the Victorian Node of the Australian National Fabrication Facility, 151 Wellington Rd., Clayton, 3168 Vic Australia

## Abstract

Nowadays, optical tweezers have undergone explosive developments in accordance with a great progress of lasers. In the last decade, a breakthrough brought optical tweezers into the nano-world, overcoming the diffraction limit. This is called plasmonic optical tweezers (POT). POT are powerful tools used to manipulate nanomaterials. However, POT has several practical issues that need to be overcome. First, it is rather difficult to fabricate plasmonic nanogap structures regularly and rapidly at low cost. Second, in many cases, POT suffers from thermal effects (Marangoni convection and thermophoresis). Here, we propose an alternative approach using a nano-structured material that can enhance the optical force and be applied to optical tweezers. This material is metal-free black silicon (MFBS), the plasma etched nano-textured Si. We demonstrate that MFBS-based optical tweezers can efficiently manipulate small particles by trapping and binding. The advantages of MFBS-based optical tweezers are: (1) simple fabrication with high uniformity over wafer-sized areas, (2) free from thermal effects detrimental for trapping, (3) switchable trapping between one and two - dimensions, (4) tight trapping because of no detrimental thermal forces. This is the NON-PLASMONIC optical tweezers.

## Introduction

Recently, there has been rapid development in plasmonics^1–5^. The electric-field-enhancement effect of plasmon can be employed as an optical force to trap a small nano/micro-object with great efficiency. It is called plasmonic optical nano-tweezers (POT)^6–9^. Most POT realisations are based on “gap-mode” surface plasmons, with which the electric field of the incident light (and accordingly the optical force) can be amplified by a factor of >10^3^ ^10^. While POT is a powerful tool with number of advantages compared with conventional optical tweezes^11–14^, there are some obstacles to further development. First, it is rather difficult to fabricate such plasmonic nanogap structures (i.e., the plasmonic dimer geometry) with high reproducibility and uniformity over large areas at low cost. Electron beam lithography^10,15,16^ is forbiddingly expensive and angular-resolved nanosphere lithography (ARNSL)^17–19^ needs complicated procedures. Second, gold and silver, which are the typical materials used in plasmonics, are expensive and increase number of processing steps for POT devices. Thus, these limitations will possibly hinder the further development of POT.

Furthermore, we and other groups previously showed that local heat generation accompanying POT cannot be regarded as negligible and that it acts as a repulsive force seriously affecting POT^10,20–23^. Although the temperature rise (*ΔT*) by plasmon excitation is not large, several tens of Kelvins frequently generate a huge temperature gradient ($\nabla T$) around the area: $\nabla T$≈1 K/μm. Such a huge temperature gradient leads to transport of nanoparticles and molecules along the temperature gradient (thermophoresis)^24–26^. In addition, thermal convection (Marangoni flow) always takes place in such situations, making the trapping behavior complex. Thus, suppression and control of the thermal forces are indispensable if POT is to progress.

Here, we present an alternative approach using a nano-structured material that can enhance the optical force. This approach uses a metal-free black silicon (MFBS) made by plasma etching^27–32^. Generally the surface of black silicon (BS) is completely covered with nano-protrusions. The optical reflection of BS is only a few percent over visible and near-IR spectral ranges due to the refractive index gradient around pyramidal nano-spikes^28^. Based on this unique non-reflecting BS platform, hence, enhanced absorbance, BS has the function of light harvesting and was used to produce high efficiency >21% solar cells^30–32^. One of the great advantages of BS is that the fabrication procedure for the nanotextured surfaces can be extended over large areas. Furthermore, we recently showed that MFBS has a small enhancement effect of electric field (***E***) of light with a factor of <5 in ***E*** ^2^ ^33^. The origin of this enhancement is due to a multiple light scattering resulting in a light localisation predominantly between the pyramidal pillars. In this study, by overcoming the listed disadvantages of POT, we demonstrate that MFBS-based optical tweezers can be used to efficiently manipulate small particles with characteristic behaviours of one and two-dimentional (1D/2D) switchable trapping and manipulation. We also showed that this optical trapping is not affected by thermal effects even at the used high intensity. This technique has various advantages, and is expected to open a new chapter in advanced optical trapping.

## Results

The trapped particles were fluorescent polystyrene nanospheres with diameters *d* = 500 nm dispersed in water. The trapping behavior was monitored using a fluorescence microscope. Prior to the optical trapping of polystyrene nanospheres using MFBS, we tested optical trapping using our conventional optical tweezers with a laser beam (808 nm laser diode) tightly focused by an oil-immersion objective lens (100 × , N.A. = 1.40). However, trapping was not stable even at the maximum output power of the laser, at which light intensity (*I*) reached 800 kW/cm^2^ at the focus. The threshold value of *I* (*I*_*tsd*_) for stable trapping was estimated to be >1000 kW/cm^2^ for our conventional optical trapping system. POT using a ARNSL substrate for these polystyrene beads has previously been investigated and *I*_*tsd*_ was evaluated to be ~ 1 kW/cm^2^ ^34^. It is obvious that the value of *I*_*tsd*_ for stable trapping is much less when the plasmonic effect is utilized.

**Figure 1:**
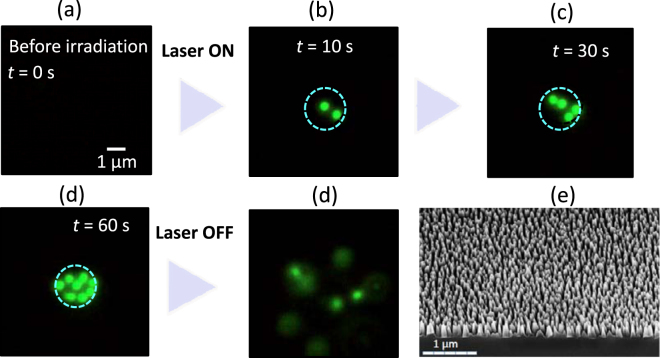
(**a**–**e**) Fluorescence micrographs of 2-dimmensional (2D) trapping behavior of polystyrene beads (*d* = 500 nm) on a metal-free black silicon (MFBS) substrate (light intensity *I* = 320 kW/cm^2^). Laser irradiation area is expressed as a blue circle (focus diameter, 3 μm). 0 s (**a**) means the time before starting laser irradiation, and the time in the images (**b**–**e**) shows laser irradiation time. Scale bar is 1 μm. (**e**) A representative scanning electron microscope (SEM) image of the MFBS surface.

We examined MFBS-assisted optical trapping of polystyrene beads using the same optical trapping system as the conventional optical tweezers and POT noted above. Figure 1(a–d) shows the results of an experiment with the polystyrene beads; a series of optical micrographs (fluorescence images) at various times, *t*, after switching on light irradiation (808 nm LD) with an intensity, *I*, of 320 kW/cm^2^ focused on the surface of the MFBS. A scanning electron microscopy (SEM) image of the MFBS used here, which was fabricated by 15 min dry etch of Si in SF_6_/O_2_ plasma, is also shown in Fig. 1(e). The height of nano-pillar was 250 nm on average and the diameter was 40–240 nm^35^. Fig. 1(b–d) clearly show trapping of polystyrene beads at the illuminated area (marked by a circular dashed-line) on the surface. The polystyrene beads were trapped one by one after the start of illumination, and the number reached 8 at *t* = 60 s. The particles were assembled on the surface of the MFBS, meaning this was a 2D-trapping. When the illumination was stopped, these trapped beads were immediately released, and spread out from the area by Brownian motion. When we replaced the MFBS with a simple glass or silicon substrate, no sign of trapping was observed at the same value of *I ﻿(irradiation intensity) ﻿*. This clearly shows the efficient optical trapping on MFBS.

A video of the above described behavior is given in the supplement information (SI) section. Although hardly apparent in the micrographs (Fig. 1), the movie revealed a unique characteristic of the trapping. In POT (see a movie in SI), the transport of a microparticle to a metallic nanostructure from some distance by Marangoni convection due to local plasmonic heating has frequently been observed^20,34,36^. However, no such transportation was observed in the present case with MFBS, confirming a thermal-effect-free character of trapping. As derived below, we estimated temperature elevation by laser irradiation on the substrates.

**Figure 2:**
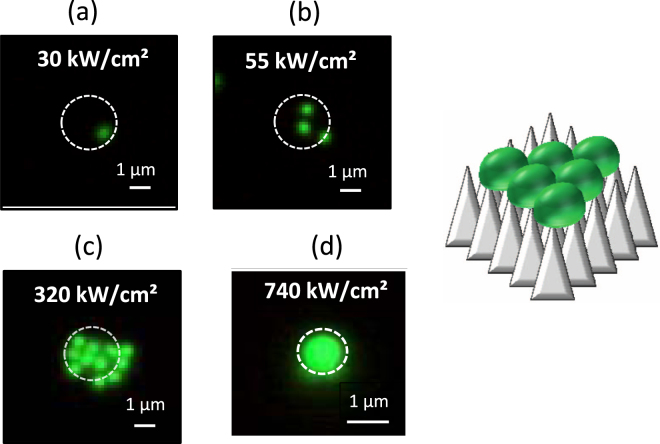
(**a**–**c**) Fluorescence micrographs of 2D-trapping of polystyrene beads (*d* = 500 nm) dependent on light intensity (*I*): (**a**) 30, (**b**) 55, (**c**) 320 kW/cm^2^. The image (**d**) at *I* = 740 kW/cm^2^ corresponds to 1D-trapping and details are noted in the Fig. 3. Irradiation area is shown as a white circle. Scale bar is 1 μm. Also, a schematic illustration of 2D-trapping of polystyrene beads on a MFBS surface is shown.

Figure 2 shows the dependence of the trapping polystyrene beads on MFBS on *I* . Below *I* = 30 kW/cm^2^, no sign of trapping was found. At *I* = 30 kW/cm^2^ (Fig. 2(a)), trapping of a single nanosphere in the irradiated area can clearly be seen. This means that the value of *I*_*tsd*_ for MFBS-assisted optical trapping is ca. 30 kW/cm^2^. This is much lower than that for conventional optical trapping (*I*_*tsd*_ ~ 1000 kW/cm^2^), and higher than that for POT (*I*_*tsd*_ ~ 1 kW/cm^2^) for the same particles^34,37–39^. That is, we were able to verify that enhancement of the optical force by MFBS greatly assists optical trapping, although the degree of enhancement is lower than that for POT.

As *I* increases, 2D-trapping with an increasing number of trapped particles can be observed on the surface of the MFBS (Fig. 2 (b,c)), indicating that the trapping potential (*U*) becomes deeper with increasing *I*. This result also signifies the important fact that optical trapping can be accomplished using a wide range of *I* from 30 to 740 kW/cm^2^ (Trapping at 740 kW/cm^2^ is 1D-trapping as described later). Note that the maximum value of *I* (740 kW/cm^2^ focused on 1 μm^2^) was the limit of our laser source and that trapping would be possible using even more intense irradiation. Such behavior is in contrast to POT, where stable trapping is possible only over a narrow range of *I*. For instance, ARNSL-based POT for the same polystyrene beads is possible only when 1 < *I* < 5 kW/cm^2^ ^34^. Stable trapping is no longer possible when *I* > 5 kW/cm^2^. This is prevented by thermal effects: thermophoresis and Marangoni convection. To reiterate, we suggest that MFBS-assisted optical trapping is free from thermal disturbance.

**Figure 3:**
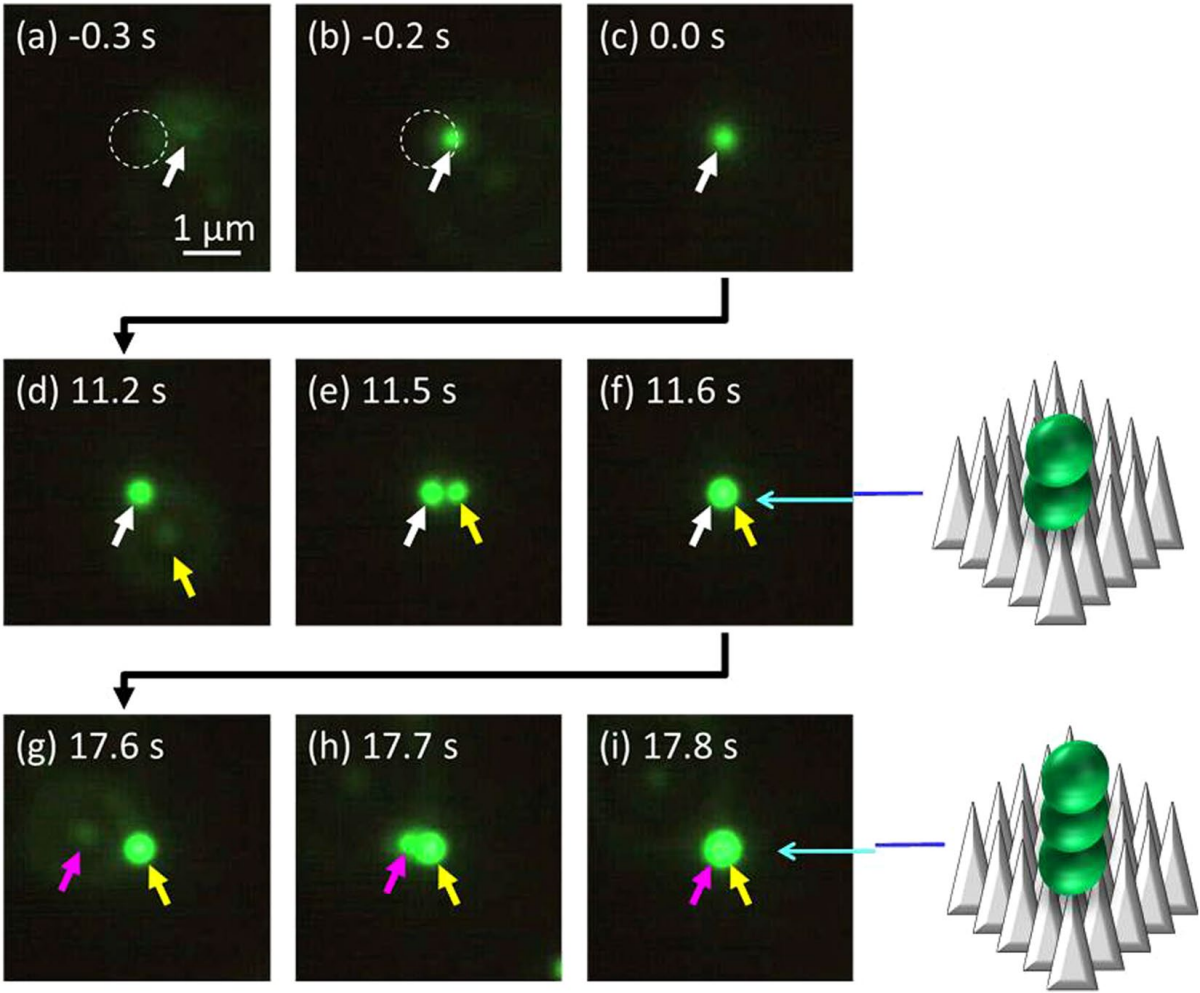
Fluorescence micrographs of 1-dimmensionally (1D) trapping behavior of polystyrene beads (*d* = 500 nm) on a MFBS substrate (light intensity *I* = 640 kW/cm^2^). Laser irradiation area (focus diameter, 1.1 μm) in the figures is smaller than that in Figs 1 and 2 (3 μm). 0 s (**c**) means the starting time of the laser irradiation. Irradiation time is given in the figure. After the first trapping event of a single particle (**a**–**c**), second particle was trapped on the top of the firstly-trapped particle (**d**–**f**). Schematic illustration shows this “snowman structure formation” by optical binding. Further continuing irradiation, the third particle was trapped on the top of the secondary-trapped particle (**g**–**i**). Schematic illustration next to (**i**) shows the 1D-trapping behavior. The image (**d**) in Fig. 2 also corresponds to the 1D-trapping.

In the aforementioned optical trapping, a 2D assembly of polystyrene nanospheres on the MFBS substrate was achieved and the site where they were trapped was circular in shape with *d* = 3 μm. Next, we show that the trapping behavior can be switched from the 2D-mode to a 1D-mode just by reducing the trapping site to 1 μm^2^. Figure 3 shows a series of optical micrographs for such trapping of polystyrene beads. In the early stage of irradiation, a single particle (marked by a white arrow in the figure) was stably trapped with no fluctuation in the illuminated area (Fig. 3(c)). When another particle (marked by a yellow arrow) happened to pass the illuminated area (Fig. 3(d,e)), it was trapped not next to the particle trapped first but on the top of it (Fig. 3(f)). This 1D trapping behavior, the “snowman structure formation” as illustrated in the figure, is shown by an increase in brightness of the fluorescence at the site (Fig. 3, from (c) to (f)).

**Figure 4:**
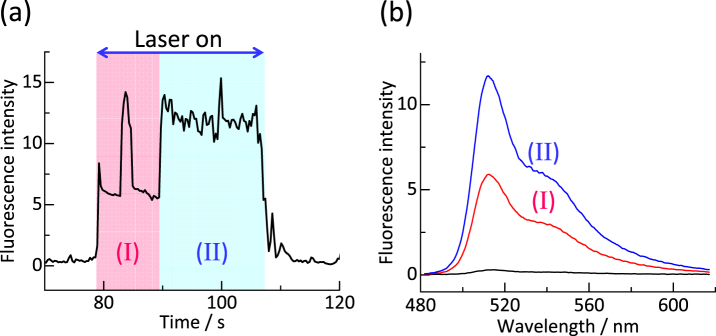
(**a**) A temporal profile of fluorescence intensity during 1D-trapping of polystyrene nanospheres (*d* = 500 nm) by laser irradiation to a MFBS substrate. This corresponds to the case of Fig. 3. 808 nm light Irradiation period consists of the area (I) and area (II). (**b**) A representative fluorescence spectrum measured at the time region (I) together with that measured at the region (II).

Upon maintaining the irradiation, a third particle (marked by a purple arrow) was trapped not next to the two particles but on top of the second trapped (Fig. 3(g–i)), as illustrated. The second or third particle were presumably fluctuate with respect to the adjacent particle, leading to the slight growth in fluorescence of the trapped particles ((c) -(f)- (i) in Fig. 3). The trapping behavior shown in Fig. 3 can more clearly be seen in the video of the supplement. Figure 4 shows a temporal profile of the fluorescence intensity (*FI*) during 1D trapping, recorded at the center of the irradiated area. It is clear that *FI* is well correlated with the trapping behavior. *FI* abruptly increases just at the start of irradiation, corresponding to the first trapped bead. After a few seconds, there is an approximately 2-fold increase in *FI* corresponding to the second trapped bead. By continuing irradiation, *FI* remains almost constant, while a third was frequently trapped during this period. The fluorescence when a third particle was trapped barely increased due to shielding of light to the particles. A vertical 1D-trapping of 3 particles along with the optical axis can be achieved, which is a peculiar and unique to MFBS-assisted trapping/binding.

Examination of all the experimental results presented here strongly implies that thermal effects are almost negligible in MFBS-assisted trapping. To explore this further, we quantitatively evaluated the temperature distribution around the excitation area during irradiation at 808 nm by means of spatially-resolved fluorescence microspectroscopy. The sample was a Tris-buffer solution of a probe dye (2′,7′-bis(2-carboxyethyl)-5-(6)-carboxyfluorescein) (BCECF), which has a fluorescence intensity very sensitive to temperature^40,41^. The fluorescence intensity of the dye decreases by 10% when the temperature increases by 15 K (see the Supplement). Using this technique, we precisely determined the rise in temperature of the irradiated area on MFBS surface. Our results showed that the fluorescence intensity of the dye was not affected by irradiation at 808 nm, indicating that the rise in temperature (*ΔT*) was negligible (*ΔT* ~ 0, and hence the gradient $\nabla T\approx 0$) over a wide range of *I* (0 < *I* < 320 kW/cm^2^). These data are also available in the Supplement. This is reasonable since crystalline silicon has an increasingly smaller optical absorption at wavelengths (λ > 800 nm) and a good thermal conductivity. Thus, we verified that the present MFBS-assisted optical trapping is completely free from thermal effects which are detrimental for a stable trapping.

## Discussion

**Figure 5:**
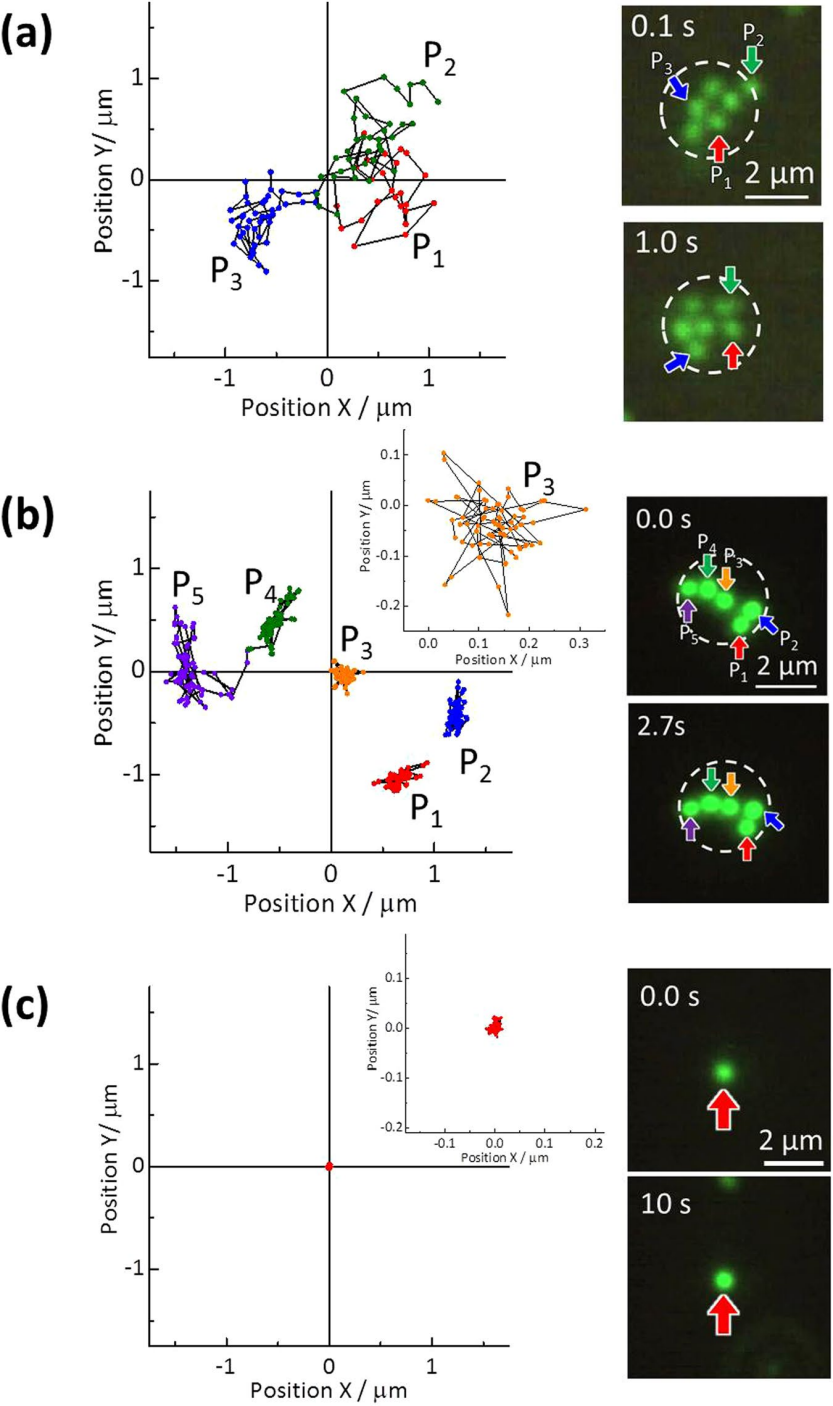
Trajectories of trapped particles as a function of time (time step, 33 ms) obtained by tracking the centroids of the particles. The monitored particles were marked by colored arrows in fluorescence micrographs. (**a**) Plasmonic optical trapping on a ARNSL surface in 1.0 s, (**b**) MFBS-assisted 2D-trapping (corresponding to Fig. 1) in 2.7 s and (**c**) 1D-trapping (corresponding to Fig. 3) in 10 s. Insets in panel (b,c) show the expanded views. From the figures, it is obvious that MFBS-assisted trapping can perform a very tight trapping. A particle is almost immobilized in Fig. 5(c).

Before we discuss the trapping mechanism, we evaluated the trap stiffness (*i.e*. how tightly the particle is trapped on the surface) on MFBS comparing with POT. Figure 5 shows trajectories of trapped particles for POT using ARNSL as a reference (Fig. 5(a))^34^ and the present MFBS-assisted trapping (Fig. 5(b) and (c), corresponding to Fig. 1 and 3, *I* = 320 kW/cm^2^). As seen in the Fig. 5(a) for POT, trapped particles were fluctuated on a ARNSL surface over ca. 1 μm within 1.0 s. This implies that trapping force is not much higher than but marginal to Brownian motion. As we previously pointed out, both the plasmon-enhanced optical force (attractive force) and the thermophoretic force (repulsive force) lay in the order of sub-pN^42^. They should cancel with each other, resulting in a small attractive force and hence the fluctuation seen in Fig. 5(a). On the other hand, it is obvious that such fluctuation was much suppressed in MFBS-assisted trapping (Fig. 5(b)). Trapped particles were fluctuated on a MFBS surface within ca. 0.5 μm. In particular, when we reduce the irradiation spot size (corresponding to Fig. 3), fluctuation of the trapped particles is negligible (Fig. 5(c)). Namely, the MFBS-assisted trapping is much tighter than POT (stiffness is strong). For this tight trapping, we can evaluate a spring constant *k* obeying Hooke’s law in the trapping potential by analyzing displacement of the particle from the center (Δ*x* or Δ*y*). As explained in the supplement, we obtained the value of *k* = 67 pN/nm at 1 W light intensity. This value is considerably larger than that of conventional optical tweezers (it ranges in the order of 10^−6^−10^﻿0﻿^ pN/nm for submicron~micron-sized polymer beads). This means that stiffness of the MFBS trapping is much high (a tight trapping). Such tighter grip would also contribute to the 1D-trapping.

**Figure 6:**
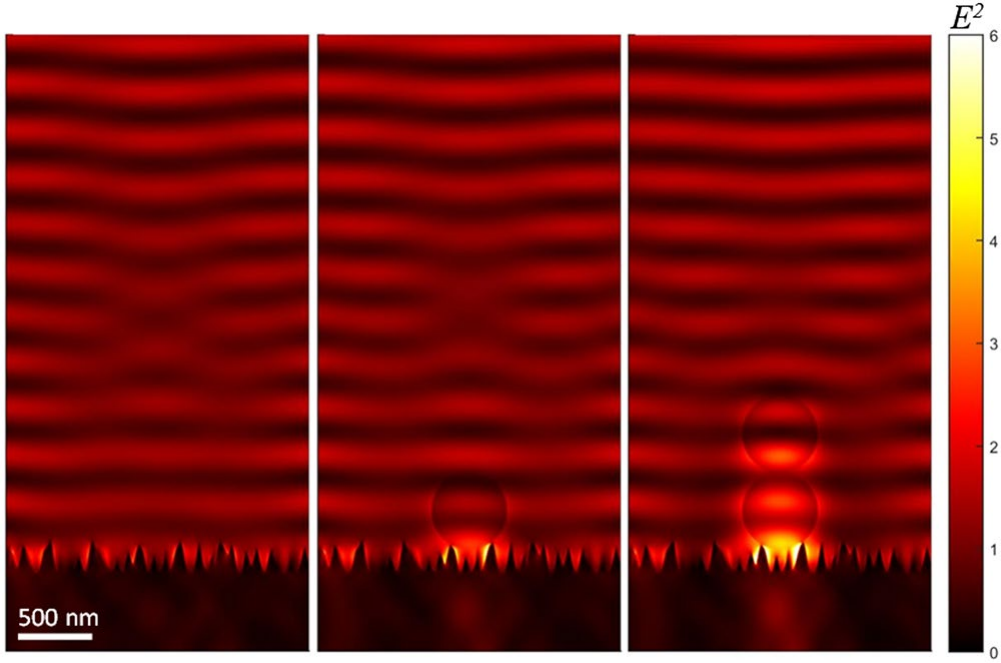
FDTD Simulation of the spatial distribution of electric field around MFBS surface irradiated with 808 nm light. (**a**) In the absence of polystyrene bead. (**b**) In the presence of polystyrene bead (single bead). (**c**) In the presence of polystyrene beads (1D-trapping of a snowman structure).

Next, the mechanism underlying the demonstrated trapping is discussed. The origin of the MFBS-assisted optical trapping is an effect of multi-scattering of the incident light which is additionally enhanced in the case of close proximity of a bead. The spatial distribution of electric field intensity of light around the surface of MFBS was calculated in the absence and presence of the polystyrene beads. Figure 6 shows the results of finite-difference time-domain (FDTD) simulation. In the absence of a particle (Fig. 6(a)), the enhancement effect is very small and is limited to the surface of the MFBS. By contrast, when the particle is placed on the surface, the electric field intensity (***E*** ^2^) is significantly enhanced at the contact location (Fig. 6(b)). This strongly suggests that the origin of the enhancement is analogous to SIBA (self-induced back action)^39,43,44^ observed in plasmonic trapping. The SIBA effect would result in the stable trapping seen in Figs. 1~3. The enhanced electric field around a trapped particle is modified^45^, and can facilitate a 1D-trapping, the tendency recognizable in Fig. 3. Figure 3(c) shows a simulation result for 1D-trapping - a snowman structure. The electric field is slightly enhanced at the interface between two particles. It is also revealed that the first particle is stronger gripped on the MFBS surface by the presence of the second particle. This SIBA-like action contributes to the tight 1D-trapping – the snowman structure.

It should be noted, that the presented FDTD modeling is for a plane wave illumination and it is not capturing focusing as well as nonlinear effects which begin to be important at high intensities; also, optical aberrations strongly dependent on the actual optics used and usually favors axially elongated focal volumes. Since polystyrene has a slightly larger refractive index than water, any bead entering 1D trapping geometry will facilitate to create light concentration due to locally augmented refractive index. This explains qualitatively well the observed 2D and 1D trapping.

Finally, we describe advantages of MFBS-assisted optical trapping. It is easy to fabricate MFBS as compared to plasmonic nanogap structures. Accordingly, rapid fabrication over a large area is possible at much lower cost than plasmonic structures. Since MFBS-assisted optical trapping is never accompanied by real electronic excitation of silicon when λ > 800 nm, it is free from thermal effects and does not suffer from thermophoresis and Marangoni convection. Thus, while POT is possible only over a narrow range of intensities *I* (1–5 kW/cm^2^ for 500 nm polystyrene beads for POT using an AR-NSL substrate^34^) due to these thermally induced localized flows, a more intense irradiation is available for MFBS-assisted optical trapping (MW/cm^2^) and opens a unexplored area of material behavior in strongly localized high intensity light fields. Such situation would enable a nonlinear optical trapping^46^ and new applications. Furthermore, while the wavelength of the light for POT is limited to a resonant plasmon band, a wide wavelengths spectrum can be used for MFBS-assisted optical trapping because anti-reflection action of tapered pyramids is inherently broadband. The switchable trapping between 1D- and 2D-trapping is also a unique feature of MFBS-assisted optical trapping.

On the other hand, MFBS has been believed to show no enhancement effect for optical trapping. It should be noted that Kotsifaki *et al*. has very recently reported POT using metal-coated BS (MCBS) (metal; gold)^47^. MCBS is an intriguing novel plasmonic material which is easy to fabricate^35,48,49^. The plasmonic functions of MCBS such as surface-enhanced Raman scattering (SERS) have been examined by several research groups, including ours^35,48^. Kotsifaki *et al*. fabricated black-Si on a Si wafer by means of femtosecond pulsed laser processing^50,51^. They quantitatively analysed trapping force and thermal effect on MFBS substrate, and showed that trapping efficiency for 400 nm polystyrene beads on the MFBS substrate was one order of magnitude higher than that on the flat silicon substrate^50^. Furthermore, optical trapping efficiency became higher by coating Au/Cu on the MFBS substrate, indicating evanescent plasmonic enhancement. Recently, they developed MCBS(gold-coated black silicon) assisted optical trapping, showing wavelength-dependent characterization of the trapping^47^. They fabricated BS by means of ultrafast (fs) laser processing which provides a slightly different morphology with rounded tips, while we employed dry etching which results in nano-sharp pyramidal surface texture. This indicates performance of MFBS-assisted optical trapping is sensitive to the actual nanostructure of silicon surface.

In conclusion, we succeeded in the MFBS-assisted optical trapping and binding of polymer nanospheres. The origin of this trapping phenomenon is multiple scattering and a SIBA-like effect which is demonstrated for dielectric (non-plasmonic) structure for the first time. MFBS-assisted optical trapping and binding have several advantages as compared with POT. Moreover, although MFBS-assisted trapping has a weaker optical force as compared to POT, MFBS-assisted trapping can perform tighter trapping than POT because it is free from the detrimental thermal effects. Namely, the total trapping force of MFBS-trapping should be higher than that of POT. By optimizing the surface nanostructure it should be possible to realize stiffer traps optimized for specific wavelengths. MFBS-assisted optical trapping mean that it can potentially be developed and applied to the manipulation of a variety of nanomaterials. MFBS-assisted optical trapping and POT should play complementary roles in the future manipulation of nanomaterials and research of nanoscale temperature and optical nonlinearities.

## Methods

The experimental methods are described in the Supplement information. Briefly, observation of POT was carried out using a fluorescence microscope, the details of which have previously been described elsewhere^34,37,42,52^. Near-infrared (NIR) laser light (λ = 808 nm) and visible laser light were used for trapping and fluorescence excitation, respectively. These laser beams were introduced co-axially into an inverted optical microscope to irradiate the sample solution. We carried out optical trapping on a surface of a substrate, since trapping force decreased of large distance from the surface^50^. Fluorescence from the dye solution was detected with a photodetector (a spectrometer with a cooled CCD camera). All the experiments were carried out at room temperature under ambient conditions.

## Electronic supplementary material

Media 1

Media 2

Media 3

Supplementary Information
