# Supplementary material for: Optical tweezing and binding at high irradiation powers on black-Si
Source: Sci Rep. 2017 Sep 26;7:12298. doi: 10.1038/s41598-017-12470-9 (PMC5614913; doi:10.1038/s41598-017-12470-9)
Supplement: Supplementary file 4 — Supplementary Information [file 41598_2017_12470_MOESM4_ESM.doc]

**Supplementary Information**

Optical tweezing and binding at high irradiation powers

on black-Si

Tatsuya Shoji^1^, Ayaka Mototsuji^1^, Armandas Balčytis^2^, Denver Linklater^2^,

Saulius Juodkazis^2,3^, and Yasuyuki Tsuboi^1^*

*^1^Graduate of Molecular Materials Science, Graduate School of Science,*

*Osaka City University, 3-3-138 Sugimoto, Sumiyoshi, Osaka, 5558-8585, Japan.*

*^2^ Centre for Micro-Photonics, Swinburne University of Technology, John Street, PO Box 218, Hawthorn, Victoria , 3122, Australia.*

^3^ *Melbourne Centre for Nanofabrication, the Victorian Node of the Australian National Fabrication Facility, 151 Wellington Rd., Clayton 3168 Vic, Australia.*

**Methods**

The experimental setup is detailed in SFig. 1. A cw near-infrared (NIR) laser (λ = 808 nm, Shanghai Laser & Optics Century Co., Ltd.) for optical trapping of polystyrene beads was coaxially introduced into a fluorescence inverted microscope (Nikon, Eclipse Ti-U) with a cw visible laser (λ = 473 nm, 3.0 μW, Shanghai Dream Lasers Co, Ltd.). These laser beams were focused on a metal-free black silicon (MFBS) substrate (a fabrication method described later) with an oil-immersion objective lens (Nikon, CFI Plan Apo VC 100×, N.A.=1.40). The area of 808 nm laser was controlled by a beam expander. The MFBS substrate set to inverted orientation. Fluorescence micrographs of polystyrene beads (diameter = 500 nm, FluoSpheres® F8813) were observed with a mercury lamp combined with the microscope. For fluorescence microspectroscopy, a polychromater (600 grooves/mm, Princeton Instruments, SpectraPro 2300) combined with a cooled charge coupled device were used. Particle positions were determined at each frame of a video (30 fps) using particle tracking software. Since the laser power of the visible laser was very weak in comparison with NIR laser, we observed no sign of optical trapping and any photobleaching by using the visible laser irradiation.

The details of a fabrication method for MFBS substrate have been already described in elsewhere.^1–3^ A silicon wafer was used as supplied. Pyramidal nano-spikes with a sub-micrometer height were formed randomly on the surface by using a lithography-free reactive ion etching (RIE) process. The aspect ratio of each spike can be controlled in the range of 1-to-10 with the dry etching parameters (*e.g.* etching time) in SF_6_/O_2_ plasma. For optical trapping in the present study, we used a MFBS substrate fabricated by 15 min dry etching time.

**SFig. 1** Optical setup for optical trapping of polystyrene beads (diameter 500 nm) on a metal-free black silicon (MFBS) substrate.

**Local temperature elevation on MFBS surface**

For estimating temperature elevation at the MFBS surface by NIR laser irradiation (λ = 808 nm), we used a temperature sensitive fluorescent dye 2’-7’-bis(2-carboxyethyl)-5(6)-carboxyfluorescein (BCECF) aqueous solution.^4,5^ Fluorescence intensity of the dye decreases with increasing temperature. Such temperature response of the dye was calibrated with a temperature control chamber. Fluorescence intensity of the dye is 10% decreased by temperature elevation of 15 K (SFig. 2(a)).

As a reference, SFig.2(b) shows fluorescence spectra of BCECF on our plasmonic (angular-resolved nanosphere lithography, ARNSL) substrate during near-infrared laser (λ = 808 nm) irradiation. Increasing light intensity of the laser (7 ~ 24 kW/cm^2^), the fluorescence intensity dramatically decreased because of local heat generation via Joule heating from the excited plasmonic nanostructures. We have already discussed the temperature elevation around the excitation, leading to the generation of a huge temperature gradient.^6^

SFig. 2(c) shows a representative fluorescence spectra of BCECF at the MFBS surface during NIR laser irradiation (light intensity = 30 and 320 kW/cm^2^) together with that without NIR laser irradiation as a reference. In contrast to the results of ARNSL substrate, the fluorescence intensity was hardly changed with NIR laser irradiation, indicating that temperature elevation was almost negligible. Therefore, we concluded that MFBS-assisted trapping does not suffer from the thermal effects including thermal convection and thermophoresis.

**SFig.3** (a) Fluorescence spectra of BCECF at different solution temperature, (b) Light intensity dependence of the fluorescence spectra on a ARNSL substrate, and (c) that on a MFBS substrate.

**Stiffness Evaluation**

SFig 4 shows a histogram of the displacement of a trapped bead in 1D-trapping at 6.4 mW of incident laser light (corresponding to 640 kW/cm^2^). The histogram was fitted by a Gaussian function defined by the root mean square displacement σ_Δ_*_x_*. The trap stiffness *k* was obtained from σ_Δ_*_x_ =* (*k_B_T*/*k*)^1/2^ (*k_B_*: Boltzman constant, *T*: temperature), being estimated to be 67 pN/nm/W. STable 1 shows the representative trap stiffness in the plasmonic optical trapping. Comparing k of MFBS-trapping with plasmonic optical trapping, our novel trapping method would strongly grip nanospheres.

**SFig.4** A histogram of displacement Δ*x* and their Gaussian fits of a first trapped polystyrene nanosphere (diameter = 500 nn, corresponding to Fig.3).

**Stable 1** Trap stiffness *k* of a trapped polystyrene nanospheres based on plasmonic optical trapping.

| Authors | Diameter of polystyrene  *d* [nm] | Trap stiffness  *k* [pN/nm/W] | Ref. |
| --- | --- | --- | --- |
| MFBS-trapping  (this study) | 500 | 67 |  |
| K. Sasaki *et al.* | 100 | 4 | [7] |
| R. Quidant *et al.* | 100 | 9.3 | [8] |
| K. B. Crozier *et al.* | 310 | 7.6 | [9] |
| K. C. Toussaint *et al.* | 1200 | 14 | [10] |

**Movies of trapping**

As other supplementary information, videos of 2D- and 1D-trapping of polystyrene beads (diameter 500 nm) with a MFBS substrate and 2D-trapping of them with a ARNSL substrate were available online.

A supplementary video (Media 1) shows 2D (transverse) trapping of the beads on a MFBS substrate at 320 kW/cm^2^ of laser irradiation (corresponding to Fig. 1 in the manuscript). Media 2 shows 1D (lateral) trapping of the beads on it (corresponding to Fig. 3 in the manuscript). Media 3 shows plasmonic optical trapping (POT) of the beads on a ARNSL substrate (corresponding to Fig. 5(a) in the manuscript) during 5 kW/cm^2^ of laser irradiation (λ = 808 nm). We have already reported the POT in the literature.^11^

**References**

1. Žukauskas, A. *et al.* Black silicon: substrate for laser 3D micro/nano-polymerization. *Opt. Express* **21,** 6901 (2013).

2. Gervinskas, G. *et al.* Surface-enhanced Raman scattering sensing on black silicon. *Ann. Phys.* **525,** 907–914 (2013).

3. Komatsu, R. *et al.* Plasmonic photo-thermoelectric energy converter with black-Si absorber. *Sol. Energy Mater. Sol. Cells* **143,** 72–77 (2015).

4. Garcés-Chávez, V. *et al.* Extended organization of colloidal microparticles by surface plasmon polariton excitation. *Phys. Rev. B* **73,** 85417 (2006).

5. Duhr, S., Arduini, S. & Braun, D. Thermophoresis of DNA determined by microfluidic fluorescence. *Eur. Phys. J. E* **15,** 277–86 (2004).

6. Toshimitsu, M. *et al.* Metallic-Nanostructure-Enhanced Optical Trapping of Flexible Polymer Chains in Aqueous Solution As Revealed by Confocal Fluorescence Microspectroscopy. *J. Phys. Chem. C* **116,** 14610–14618 (2012).

7. Tanaka, Y., Kaneda, S. & Sasaki, K. Nanostructured potential of optical trapping using a plasmonic nanoblock pair. *Nano Lett.* **13,** 2146–50 (2013).

8. Juan, M. L., Gordon, R., Pang, Y., Eftekhari, F. & Quidant, R. Self-induced back-action optical trapping of dielectric nanoparticles. *Nat. Phys.* **5,** 915–919 (2009).

9. Wang, K., Schonbrun, E., Steinvurzel, P. & Crozier, K. B. Trapping and rotating nanoparticles using a plasmonic nano-tweezer with an integrated heat sink. *Nat. Commun.* **2,** 469 (2011).

10. Roxworthy, B. J. & Toussaint, K. C. Femtosecond-pulsed plasmonic nanotweezers. *Sci. Rep.* **2,** 660 (2012).

11. Shoji, T. *et al.* Reversible Photoinduced Formation and Manipulation of a Two-Dimensional Closely Packed Assembly of Polystyrene Nanospheres on a Metallic Nanostructure. *J. Phys. Chem. C* **117,** 2500–2506 (2013).
